# Constitutive expression of Bcl-2 induces epithelial-Mesenchymal transition in mammary epithelial cells

**DOI:** 10.1186/s12885-015-1485-5

**Published:** 2015-06-20

**Authors:** Juan An, Jin Lv, Aimin Li, Junxiao Qiao, Liang Fang, Zhihua Li, Bo Li, Wei Zhao, Huoming Chen, Liying Wang

**Affiliations:** 1 The Second Artillery General Hospital, PLA, Beijing, 100088 China; 2 Department of Hepatobiliary Surgery, The Second Artillery General Hospital, Beijing, 100088 China

**Keywords:** Bcl2, Epithelial, Mesenchymal, Differentiation

## Abstract

**Background:**

Bcl-2 (B cell lymphoma/leukemia gene-2) is the first proto-oncogene recognized to function by inhibiting programmed cell death/apoptosis. Although much is known about the anti-apoptotic ability of Bcl-2, little information is available concerning its function in other cellular processes, such as cell differentiation.

**Methods:**

In this study, stable cell lines from pre-malignant MCF10ATG3B mammary epithelial cells, a cell line derived from a human proliferative breast disease model, to express exogenous Bcl-2 was established. CMV promoter driven Bcl-2 expression vector or empty vector was transfected into MCF10ATG3B human mammary epithelial cells to investigate the effects of Bcl-2 on mammary epithelial cells. In addition, western blot and immunofluoresence staining were employed to testify the marker proteins of both mesenchymal and epithelial cells.

**Results:**

Unexpectedly, a dramatic change of phenotype from epithelial cells to fibroblast-like cells was observed in Bcl-2-transfected cells. Western blot analysis and immunofluoresence staining results demonstrated that the E-cadherin and desmoplakin, markers of epithelial cells, were downregulated in the Bcl-2-transfected cells. However, N-cadherin and vimentin, markers of mesenchymal cells, were upregulated in these cells. Redistributions of cytokeratin and beta-catenin were also observed in the Bcl-2-transfected cells. Our results further showed that the Bcl-2-transfected MCF10ATG3B cells retained some epithelial markers, such as epithelial specific antigen (ESA) and epithelial membrane antigen (EMA), indicating their epithelial origin. In addition, cell migration and invasion was substantially increased in Bcl-2 transfected cells.

**Conclusion:**

Taken together, our results strongly indicate that in addition to its anti-apoptotic function, Bcl-2 is also involved in the epithelial-mesenchymal transition (EMT), a fundamental mechanism in normal morphogenesis and pathogenesis of some diseases.

## Background

Proto-oncogene Bcl-2 (B cell lymphoma/leukemia gene-2) is a main member of Bcl-2 family, which plays a central role in regulation of programmed cell death pathways by suppressing (*e.g.* Bcl-2, Bcl-XL) or promoting (*e.g.* Bax, Bak, Bad, Bcl-Xs) apoptosis. It has been demonstrated that Bcl-2 expression is required for long-term cell survival or cell transformation [1–3]. Bcl-2 inhibits apoptosis induced by a variety of stimuli including tumor necrosis factor (TNF), cytotoxic drugs, and ionizing radiation [1–3]. Although much is known about the anti-apoptotic ability of Bcl-2, little information is available concerning its functions in other cellular processes, such as cell differentiation.

Proliferation, differentiation, and apoptosis are processes tightly regulated during development and tissue homeostasis, allowing amplification along specific lineages while preventing excess proliferation of immature cells. Dysregulation of these processes contributes to some diseases including cancer. Epithelial cell adhesion and communication with the extracellular matrix (ECM) and neighboring cell play fundamental roles in epithelial trans-differentiation into a mesenchymal phenotype which involves in some stress kinases, phosphatase2A, and phosphositide 3-kinase (PI3-kinase)/protein kinase B (AKT) [4–7], which share some similar signal transduction pathways with apoptosis regulation pathways of Bcl-2 family.

In this study, we showed that the constitutive expression of Bcl-2 in human mammary epithelial MCF10ATG3B cells induced epithelial-mesenchymal transition (EMT). Our results thus indicate that in addition to its anti-apoptotic function, Bcl-2 may be also involved in EMT during normal morphogenesis and tumorigenesis.

## Methods

### Antibodies

Antibodies against E-cadherin, N-cadherin, α-catenin, β-catenin and γ-catenin were purchased from BD Science Transduction Laboratories (Lexington, KY, USA). Antibodies against Desmoplakin I&II, vimentin (AB-2), Epithelial Specific Antigen (Ab-2) and Epithelial Membrane Antigen (Ab-2) were obtained from LabVision Corporation (Fremont, CA, USA). The β-actin antibody, the goat anti-mouse IgG-HRP, the goat anti-rabbit IgG-HRP and the donkey anti-goat IgG-HRP antibodies were purchased from Santa Cruz Biotechnology (Santa Cruz, CA, USA). Anti-rabbit and mouse Alexa Fluor 488 antibodies were purchased from Invitrogen Life Technologies (Grand Island, NY, USA).

### Cell culture and DNA transfection

MCF10AT3B epithelial cells were obtained from Karmanos Cancer Institute (Detroit, MI, USA) [8]. MCF10AT3B cells and its derivatives were maintained at 37 °C in a 5 % CO_2_ atmosphere in DMEM/F12 supplemented with 5 % horse serum, L-Glutamine (2 mM), penicillin (100 U/ml), streptomycin (100 μg/ml), hydrocordisone (0.5 μg/ml), insulin (10 μg/ml), Epidermal growth factor (EGF) (2 ng/ml), and clolera toxin (0.1 μg/ml). For DNA transfection, cells were plated at a density of 1 × 10^5^ per 60-mm dish and transfected 24 h later with a pcDNAI-Bcl-2 expression vector driven by the cytomagalovirus (CMV) promoter (kindly provided by Dr. HR-C Kim at Wayne State University) as described before [9]. using FuGene6 transfection reagent (Promega, Madison, WI, USA) according to the manufacture’s instruction. DNA transfection was performed with 15 μg of linearized expression vector and 5 mg of an expression vector containing G418 resistant marker gene. The empty expression vector was used as a control. Forty-eight hours after transfection, the cells were re-plated and selected with 500 μg/ml of G418 (Invitrogen Life Technologies). The medium was changed every three days until colonies appeared. Individual single colonies were then isolated and expanded to confirm expression of Bcl-2 by Western blot analysis.

### Western blot analysis

Cells were washed with cold phosphate buffer saline (PBS) and lysed with the radio-immunoprecipitation assay (RIPA) buffer containing 1 % proteinase inhibitor cocktail solution and 1 % phosphatase inhibitor cocktail solution (Sigma, St. Louis, MO, USA). The cell lysates were boiled for 5 min in sodium dodecyl sulfate (SDS) gel-loading buffer and separated on 10 % SDS-PAGE gels. After electrophoresis, the proteins were transferred to a polyvinylidene fluoride (PVDF) membrane (Bio-Rad Laboratories, Hercules, CA). The membranes were probed with appropriate primary antibodies and visualized with the corresponding secondary antibodies and the electro-chemi-luminescence (ECL) kit (Thermo Scientific, Rockford, IL, USA). The same membranes were stripped and re-probed with an antibody for β-actin to confirm equal loadings.

### Indirect immunofluorescence staining

Cells were fixed in 4 % paraformaldehyde for 10 min, then permeabilized in 0.1 % Triton X-100 for 5 min, blocked in 1 % BSA for 30 min, and then incubated with primary antibodies at 4 °C overnight. Secondary antibodies, anti-rabbit or mouse Alexa Fluor 488 was then added and incubated for 1 h at room temperature. Cells were washed with PBS and mounted with 10 mg/ml DAPI (4, 6-diamidino-2-phenylindole dihydrochloride) (Sigma-Aldrich, St. Louis, MO, USA) in aqueous mountant (Dako, Carpinteria, CA, USA) and photographed using a fluorescent Nikon microscope at a 63× magnification. Images were captured using the MRC-1024 confocal Imaging System (Bio-Red, Hercules, CA, USA).

### Cell viability assay

To determine cell viability, 5 × 10^4^ of cells were seeded in triplicate onto 35-mm dishes, treated with different concentrations of serum, hydrocordisone withdrawal, and different concentrations of TNF (R & D systems, Minneapolis, MN, USA) for five days, then trypsinized and the cell viability was determined with the ADAM automatic cell counter (Digital Bio. Hopkinton, MA, USA) according to the manufacture’s recommendation.

### Migration and invasion assay

The cells were plated at a density of 1 × 10^6^ per 60-mm dish, incubated for 24 h, and drew a line with a sterilized pipette tip on the dish bottom to take off the cells, then changed to fresh medium. After incubation for another 24 h, cell migration was examined and photographed.

To measure invasion ability of these cells, we used a 24-well BD BioCoat Matrigel Invasion Chamber (BD Biosciences, Bedford, MA, USA). An equal number of cells (2.5 × 10^4^/ml) in 0.5 ml medium were loaded into the top chamber of each well. After incubating the chamber for 24 h, non-migrating cells were scraped from upper surface of the filter. The cells on lower surface were fixed with ethanol, stained with Diff-Quick Stain Set (Andwin Scientific Direct, Schaumburg, IL, USA), and photographed.

### Statistical analysis

Data were summarized as the mean ± standard error (SE) using the GraphPad InStat software program (GraphPad Software, La Jolla, CA, USA). Tukey-Kramer Multiple Comparisons Test was used, and the significance was accepted for *p* < 0.05.

## Results

### Constitutively expression of Bcl-2 in MCF10ATG3B cells attenuates apoptosis induced by serum starvation, hydrocordisone withdrawal and TNF-a treatment

**Fig. 1:**
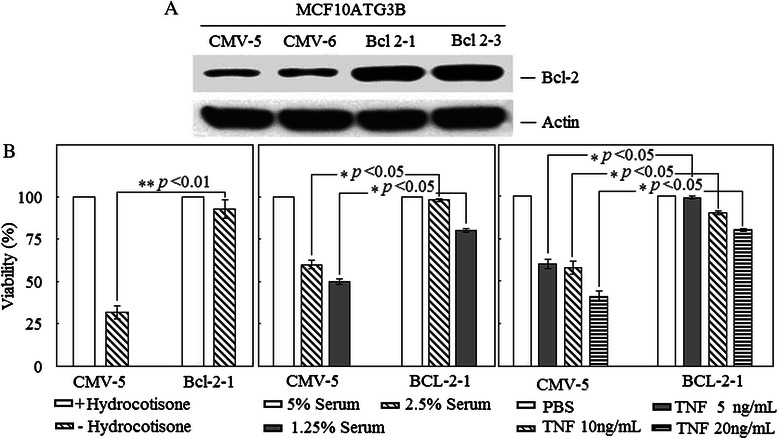
Effects of constitutive Bcl-2 expression on the morphology of MCF10ATG3B cells. **a** Western blot analysis of the cell lysates from Bcl-2-transfected cells (Bcl-2-1 and −3) and control cells transfected with empty expression vector (CMV-5 and −6) using an anti-Bcl-2 specific antibody and an anti-actin antibody to ensure an equal loading. **b** BCl-2-1 and CMV-5 cells were cultured in different concentrations of serum as indicated for three days, or cultured in medium with or without hydrocordisone for two days, or treated with different concentrations of TNF for two days. Cell survival assays were performed as described in Methods

To investigate the effects of Bcl-2 on mammary epithelial cells, we transfected MCF10ATG3B human mammary epithelial cells either with a CMV promoter driven Bcl-2 expression vector or with an empty vector, and selected transfectants with G418 for three weeks. The cell colonies were isolated and expanded. The expression of Bcl-2 in the clonal cell lines was then evaluated with Western blot analysis. Our results showed that Bcl-2 was highly expressed in the Bcl-2-transfected cells compared with control cells transfected with the empty vector (Fig. 1a). We also observed endogenous Bcl-2 expression in the control cells (Fig. 1a).

We established a number of stable cell lines that expressed Bcl-2, two of which are described in detail in this report (Bcl-2-1, −3). We also generated two control cell lines transfected with the empty vector (CMV-5, −6). The growth rate of each cell line was determined by counting the number of cells daily. There was no significant difference in the growth rate between the Bcl-2-expressing cells and control cells (data not shown). We then decided to determine whether the transfected Bcl-2 was functionally active in the transfectants by examining the effect of Bcl-2 on programmed cell death/apoptosis. First, we cultured cells in medium containing different concentrations of serum and found the viability of control cells was dramatically reduced in lower concentrations of serum; about 60 % cells were non-viable in the medium containing 1.25 % serum while Bcl-2 expressing cells were more resistant to serum starvation (Fig. 1b). It has been reported previously that hydrocordisone is vital to normal growth of MCF10A cells in culture and hydrocordisone withdrawal will induce cell apoptosis in MCF10A cells [10, 11]. We cultured cells in the absence of hydrocordisone and found that Bcl-2 expression attenuated cell apoptosis induced by hydrocordisone withdrawal (Fig. 1b). Tumor necrosis factor (TNF) is a potent inducer of cell apoptosis [6]. We found that Bcl-2 expressing cells were also resistant to TNF-induced apoptosis compared to control cells (Fig. 1b). Our data thus indicated that the constitutive expression of exogenous Bcl-2 inhibited apoptosis induced by serum starvation, TNF treatment and hydrocordisone withdrawal and suggesting that transfected Bcl-2 is functionally active in the transfected cells.

### Constitutively expression of Bcl-2 in MCF10ATG3B cells induces epithelial to mesenchymal transition

**Fig. 2:**
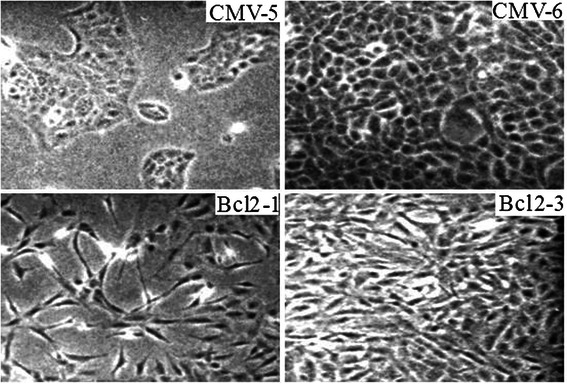
Constitutive Bcl-2 expression induces morphology change. Cell morphologies were examined in Bcl-2 transfected cells and control cells at different cell density under a microscope and photographed (magnification X 40)

Unexpectedly, we also observed a dramatic change of phenotype from epithelial cells to fibroblast-like cells in the Bcl-2-transfected MCF10ATG3B cells that exhibited a highly elongated and spindle-shaped phenotype and failed to form extensive cell-cell contacts. However, the vector transfected MCF10ATG3B cells still kept epithelial characteristic (Fig. 2).

**Fig. 3:**
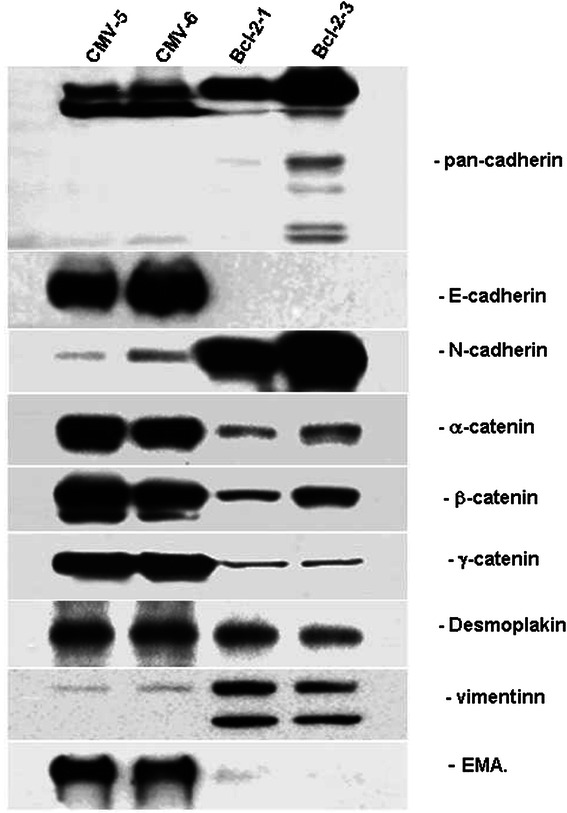
Constitutive Bcl-2 expression induces an epithelial-mesenchymal transition associated with the loss of epithelial markers and the gain of mesenchymal markers. Western blot analysis of expression levels of epithelial and mesenchymal markers in Bcl-2 transfected cells and control cells using antibodies specific to E- and N-cadherin, α, β, and γ-catenin, vimentin, and epithelial membrane antigen (EMA)

**Fig. 4:**
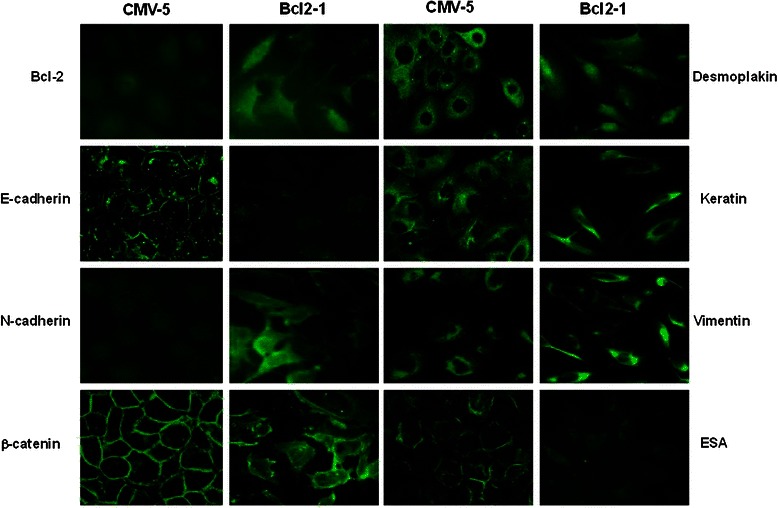
Immunofluorescent evidence of epithelial-mesenchymal trasition induced by constiutive Bcl-2 expression associated with the loss of epithelial markers and the gain of mesenchymal markers. Cells were fixed and immunofluorescent staining was performed using antibodies specific to E- and N-cadherin, β-catenin, desmoplakin I&II, vimentin and epithelial specific antigen (ESA) and FITC-conjugated secondary antibodies. The images were photographed under a Bio-Rad MRC1024 confocal scanning laser microscope (magnification X 60)

Since the Bcl-2-expressing MCF10ATG3B cells acquired a fibroblast-like morphology, we next examined the marker proteins of both mesenchymal and epithelial cells by Western blot analysis (Fig. 3) and immunofluoresence staining (Fig. 4).

Transmembrane cadherins are a family of cell-cell adhesion molecules that play a central role in tissue morphogenesis and homeostasis. Cadherin function is regulated by its association with the actin containing cytoskeleton, an association mediated by a complex of cytoplasmic proteins, the catenins: α, β and γ. We first noted that the expression profile of junctional proteins was markedly altered in the Bcl-2-expressing MCF10ATG3B cells compared with the control cells (Fig. 4). Western blot analysis demonstrated that the expression of the epithelial specific proteins, E-cadherin and epithelial membrane antigen (EMA) were almost completely downregulated in the Bcl-2-expressing cells. However, N-cadherin, a main mesenchymal marker, was highly expressed in these cells. We also observed protein bands recognized by pan-cadherin antibody were different in two Bcl-2 expressing cell lines, suggesting that relative levels of these proteins may be different in two cell lines. In accordance with Western blot results, confocal immunofluorescence microscopy revealed a membrane and also a cytoplasmic distribution of N-cadherin in the Bcl-2-expressing cells. E-cadherin and epithelial specific antigen (ESA) were still detectable above background levels in the Bcl-2-expressing cells, but disappeared from the plasma membrane and scattered in the cytoplasm. In contrast, a very strong immuno-staining of these proteins was observed on the plasma membrane in the control cells. Concomitantly, the localization of the catenins was also markedly altered. β-catenin was co-localized with E-cadherin along the entire plasma membrane in the control cells, but mainly found in the cytoplasm and appeared to be independent of E-cadherin in the Bcl-2-expressing cells. In addition, desmoplakin was detected as dot-like structures in the cytoplasm; disappeared from the plasma membrane of the BCl-2-expressing cells, indicating that desmosomal structures were internalized as large complexes upon loss of epithelial polarity. We also observed a dramatic change in the expression of vimentin, another mesenchymal marker. Western blot analysis showed that vimentin was detected as closely spaced double-band of ~44kD and ~40kD in the Bcl-2-expressing cells, but only a very weak band at ~44kD in the vector control cells. Immunofluorescence staining showed a cytoplasmic threadlike structure of vimentin in the Bcl-2-expressing cells compared a weaker staining in the control cells (Fig. 4).

### Bcl-2-overexpressing MCF10ATG3B cells display a higher migratory and invasion capability than control cells

**Fig. 5:**
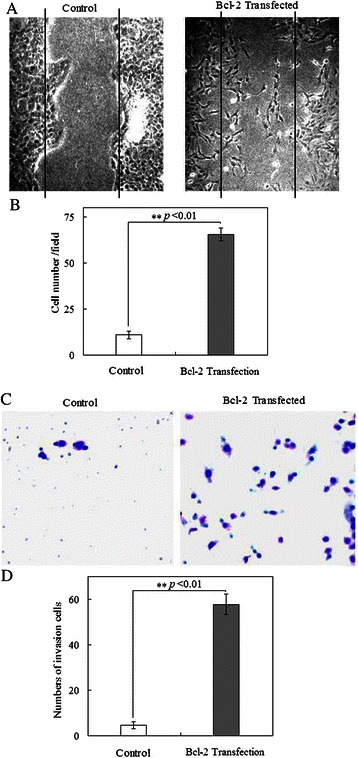
Constitutive Bcl-2 expression induces a migratory and invasive phenotype. **a** The motility behavior of control cells and Bcl-2 transfected cells was analyzed in an in vitro wound-healing assay. **b** Statistical significance (*p* < 0.01) exhibited for the Bcl-2 transfected cells with stronger migratory capability (*N* = 3; mean = 65.33; SE = 3.51) than the control ones (*N* = 3; mean = 11; SE = 2.0).. Data showed the counted cell numbers, and represented as mean ± s.e.m. **c** The invasive features of the control and Bcl-2 expressing cells were analyzed in an invasion assay on matrigel in a BioCoat Matrigel Invasion Chamber. The cells adhered to the lower surface of the filters were fixed and stained with a Diff-Quik Stain Set, photographed under a light microscope. **d** Statistical significance (*p* < 0.01) exhibited for the Bcl-2 transfected cells with stronger invasive potential (*N* = 3; mean = 57.67; SE = 4.51) than the control ones (*N* = 3; mean = 4.67; SE = 1.53)

The acquisition of an EMT phenotype prompted us to examine the migration and invasion ability of Bcl-2- expressing cells. A typical *in vitro* wound-healing assay was performed. We found that Bcl-2 expressing cells showed more rapid closure of the wound created in a two-dimensional culture compared to control cells (*p* < 0.01; Fig. 5a and b). We next tested the invasive ability of the Bcl-2 expressing cells through matrigel-coated filters and found only the Bcl-2-expressing cells invaded through matrigel, and exhibited a dramatically increased number of invading cells compared to the control cells (*p* < 0.01; Fig. 5c and d). Our results indicated a directly or indirectly involvement of Bcl-2 in the acquisition of EMT-like phenotype that paralleled the enhanced motility and invasive ability.

## Discussion

Programmed cell death or apoptosis is a highly regulated process responsible for the removal of damaged and unnecessary cells. The dynamic interplay between pro-death proteins (BAX, BAK, BAD, BIM, NOXA, and PUMA) and pro-survival proteins (BCL-2, BCL-XL, and BCL-W) controls cell apoptosis [2]. Dysregulation of the balance between these opposing proteins may result in uncontrolled cell growth and even tumorigenesis.

The tumorigenic potential of the overexpression of the anti-apoptotic gene Bcl-2 was first described as a result of the chromosomal translocation seen in subsets of non-Hodgkin’s lymphoma, where it has been found to be tumorigenic [1–3]. Enhanced expression of Bcl-2 has been identified in other malignancies, including breast cancer, where its pathological function is less clear [10, 11].

In this study, we revealed an additional function of Bcl-2; its constitutive expression has a profound morphological effect on mammary epithelial cells, MCF10AT3B. First, Bcl-2 transfected cells displayed elongated and spindle-shaped morphologies, a typical fibroblastoid phenotype. Second, these morphologic changes are associated with the loss of epithelial markers such as E-cadherin and the gain of mesenchymal markers such as N-cadherin and vimentin. Third, the adherens junctions among cells are completely diminished and all catenins and E-cadherin are downregulated and redistributed. Fourth, the Bcl-2 expressing cells acquired enhanced motile and invasive activities. All of these changes resemble a typical EMT process, which is characterized by the loss of epithelial properties and the gain of mesenchymal features [4–7].

Analysis of cellular marker protein expression indicated that the effect of constitutive Bcl-2 expression on cells is not limited to cell morphology. Constitutive Bcl-2 expression affects expression of genes that determine cellular behaviors such as the loss of epithelial cellular marker E-cadherin and the gain of mesenchymal N-cadherin. The shift in expression from E- to N-cadherin in Bcl-2 transfected cells again indicated that Bcl-2 expression induced a typical EMT. Previously, it was reported that forced expression of N-cadherin in breast cancer cells MCF-7 induces cell migration, invasion and metastasis without changes of the endogenous expression or adhesive function of E-cadherin and cell epithelial phenotypes [12], suggesting cell migration and invasion is a cellular process independent of the EMT. Thus, it is likely that the induced N-cadherin expression in Bcl-2-transfected cells is responsible for the enhanced cell motile and invasive activities observed, whereas E-cadherin downregulation contributes to the disassembly of the adherens junctions during Bcl-2 trigged EMT.

The importance of E-cadherin in maintaining epithelial phenotype is well documented [13]. The downregulation of E-cadherin expression and function is a critical step during EMT and in the malignant progression of most epithelial tumors [4]. Here, we observed that E-cadherin is downregulated and redistributed in Bcl-2 transfected cells. Currently, it is unclear how constitutive Bcl-2 expression results in loss of E-cadherin expression. The transcription repressors such as Snail [14], SIP1 (ZEB-2) [15] and Twist [16] suppress the transcription of E-cadherin gene and induce EMT. Recently, it was reported that Bcl-2 physically interacts with Twist1, which leads to transcriptional regulation of Twist target gene such as E-cadherin [17]. Thus, Bcl-2 may be directly involved in transcriptional regulation of the transcription factors involved in regulation of E-cadherin expression.

## Conclusions

Our results here are in good agreement with the previous reports of Bcl-2 function in invasion and metastasis [18–21] and that Bcl-2 over expression influences E-cadherin expression and promote EMT in different types of cells [17, 22, 23]. Moreover, gene expression profiles in human cancers indicated that de-differentiated cancer cells share EMT properties with a stem-like phenotype [24]. A direct link between EMT and cancer stem cells was demonstrated by findings that EMT activators, such as Twist1, can induce both EMT and stemness [25, 26]. Thus, it will be interesting to investigate the function of Bcl-2 induced EMT in the stemness of human breast cancer stem-like cells.

## Abbreviations

Bcl-2: B cell lymphoma/leukemia gene 2
TNF: Tumor necrosis factor
ESA: Epithelial specific antigen
EMA: Epithelial membrane antigen
ECM: Extracellular matrix
EMT: Epithelial mesenchymal transition
PBS: Phosphate buffer saline
RIPA: Radio-immunoprecipitation assay
CMV: Cytomagalovirusv
EGF: Epidermal growth factor
DNA: Deoxyribonucleic acid
PI3-kinase: phosphositide 3-kinase
AKT: Protein kinase B
SDS: Sodium dodecyl sulfate
PVDF: Polyvinylidene fluoride
ECL: Electro-chemi-luminescence
DAPI: 6-diamidino-2-phenylindole dihydrochloride
